# Differential signaling pathway activation in 7,12-dimethylbenz[a] anthracene (DMBA)-treated mammary stem/progenitor cells from species with varying mammary cancer incidence

**DOI:** 10.18632/oncotarget.25988

**Published:** 2018-08-28

**Authors:** Melissa M. Ledet, Meghan Oswald, Robyn Anderson, Gerlinde R. Van de Walle

**Affiliations:** ^1^ Baker Institute for Animal Health, College of Veterinary Medicine, Cornell University, Ithaca 14853, NY, USA

**Keywords:** mammary stem/progenitor cells, DMBA, mammary cancer, DNA damage, apoptosis

## Abstract

A natural variation exists in the susceptibility to mammary cancer among wild and domestic mammalian species. Mammary stem/progenitor cells (MaSC) represent a primary target cell for transformation; however, little is known about the intrinsic response of these cells to carcinogenic insults. Polycyclic aromatic hydrocarbons (PAH), such as 7,12-dimethylbenz[a]anthracene (DMBA), are abundantly present in the environment and have been linked to the development of mammary cancer in humans and rodents. We treated MaSC from equine (mammary cancer-resistant) and canine (mammary cancer-susceptible) species with DMBA and assessed cytochrome P450 metabolic activity, DNA damage and viability. Our notable findings were that MaSC from both species showed DNA damage following DMBA treatment; however, equine MaSC initiated cell death whereas canine MaSC repaired this DNA damage. Follow-up studies, based on genome-wide transcriptome analyses, revealed that DMBA induced activation of both the intrinsic and extrinsic apoptotic pathways in equine, but not canine, MaSC. Based on these findings, we propose a hypothetical model in which undergoing apoptosis in response to an oncogenic event might contribute to a lower incidence of mammary cancer in certain mammalian species. Such a mechanism would allow for the elimination of DNA-damaged MaSC, and hence, reduce the risk of potential tumor-initiating mutations in these cells.

## INTRODUCTION

Peto’s paradox describes the lack of correlation between body size and cancer risk [1]. Larger animals, with more cells in their body compared to smaller animals, do not have an increased cancer risk as would be expected. Elephants are one such example of a species with a greater than average body size and a less than average cancer incidence [2]. It was reported recently that these animals have additional copies of the tumor suppressor gene p53, providing a potential explanation for their cancer resistance. While humans have two p53 alleles, elephants were found to have 40 alleles [2]. Additional species for which the underlying mechanisms of cancer resistance have been studied are the naked mole rat and the blind mole rat. Both are long-lived rodents with exceptionally low cancer incidences. Naked mole rat fibroblasts were found to have early contact inhibition as a result of their ability to secrete high molecular mass hyaluronan, which mediates their cancer resistance [3, 4]. In response to pro-growth signals, blind mole rat cells trigger a necrotic cell death response, and this concerted cell death has been attributed to this species’ resistance to cancer [5].

A low incidence in certain cancers, however, is not unique to wild species. For example, the horse, which is a domesticated species, has an overall low cancer incidence (3%) despite having a large mass and relatively long lifespan [6]. Moreover, this species is unique in respect to the distribution of the cancer types that do occur. Whereas 80% of pale pigmented horses will develop melanoma by age 15, only 0.03% of mares will develop mammary cancer [7–12]. In contrast, mammary cancer is the most frequently diagnosed cancer in female dogs, another domesticated species, accounting for 70% of all cancer cases [13]. While differences in reproductive strategies and diet have been proposed to play a role in this variation of mammary cancer incidence, they alone cannot account for the striking differences in mammary cancer prevalence between ungulates, such as the horse, and carnivores, such as the dog [12].

We recently found differences in the intrinsic behavior of mammary stem/progenitor cells (MaSC) from horse and dog [14]. Specifically, we found that MaSC from these species showed a striking difference in growth potential in long-term cultures, which led to the identification of a novel form of intercellular communication involving microvesicle-mediated Wnt/β-catenin signaling [14]. Since MaSC have a unique capacity for self-renewal and persist for the lifetime of the animal [15, 16], they are thought to represent a primary target cell for the origin and development of mammary cancer [17–19]. In this regard, it would be of significant interest to study the intrinsic behavior of these cells in response to mammary-specific oncogenic insults.

Polycyclic aromatic hydrocarbons (PAH) are a class of environmental chemicals, with many being carcinogenic in both rodent models and humans [20, 21]. As products of combustion, PAH exposure most commonly occurs in air pollution, auto exhaust, smoking, diesel, and grilled or smoked foods [22, 23]. In particular, exposure and susceptibility to PAH is significantly associated with breast cancer in women [24], and the ability of the synthetic PAH 7,12-dimethylbenz[a]anthracene (DMBA) to induce mammary tumors in rodents is well documented [20]. In order to exert its carcinogenic effects, DMBA must first be metabolically activated by members of the superfamily cytochrome P450 (CYP450), such as CYP1A1 and CYP1B1 [21, 25, 26]. Once the principal ultimate carcinogenic metabolite DMBA-3,4-diol-1,2-epoxide is formed, it can bind to cellular DNA to form DNA adducts, which lead to mutations that are prerequisites of tumor development [27, 28].

The goal of the present study was to (i) evaluate the intrinsic behavior of MaSC from the mammary cancer-resistant species, the horse, in response to DMBA *in vitro* and (ii) compare this to the response of MaSC from dogs, a mammary cancer-susceptible species. Our prominent findings were that MaSC from both species showed DNA damage following DMBA treatment; however, equine MaSC initiated cell death whereas canine MaSC repaired this DNA damage. Moreover, we identified that the DMBA-induced apoptosis in equine MaSC was mediated through activation of both the intrinsic and extrinsic apoptotic signaling pathways. Based on these findings, we propose a hypothetical model in which undergoing apoptosis in response to an oncogenic event might contribute to a lower incidence of mammary cancer observed in certain mammalian species. Such a mechanism would allow for the elimination of DNA-damaged MaSC, and hence, reduce the risk of potential tumor-initiating mutations in these cells.

## RESULTS

### DMBA induces cytochrome P450 activity and DNA damage in both equine and canine MaSC

**Figure 1:**
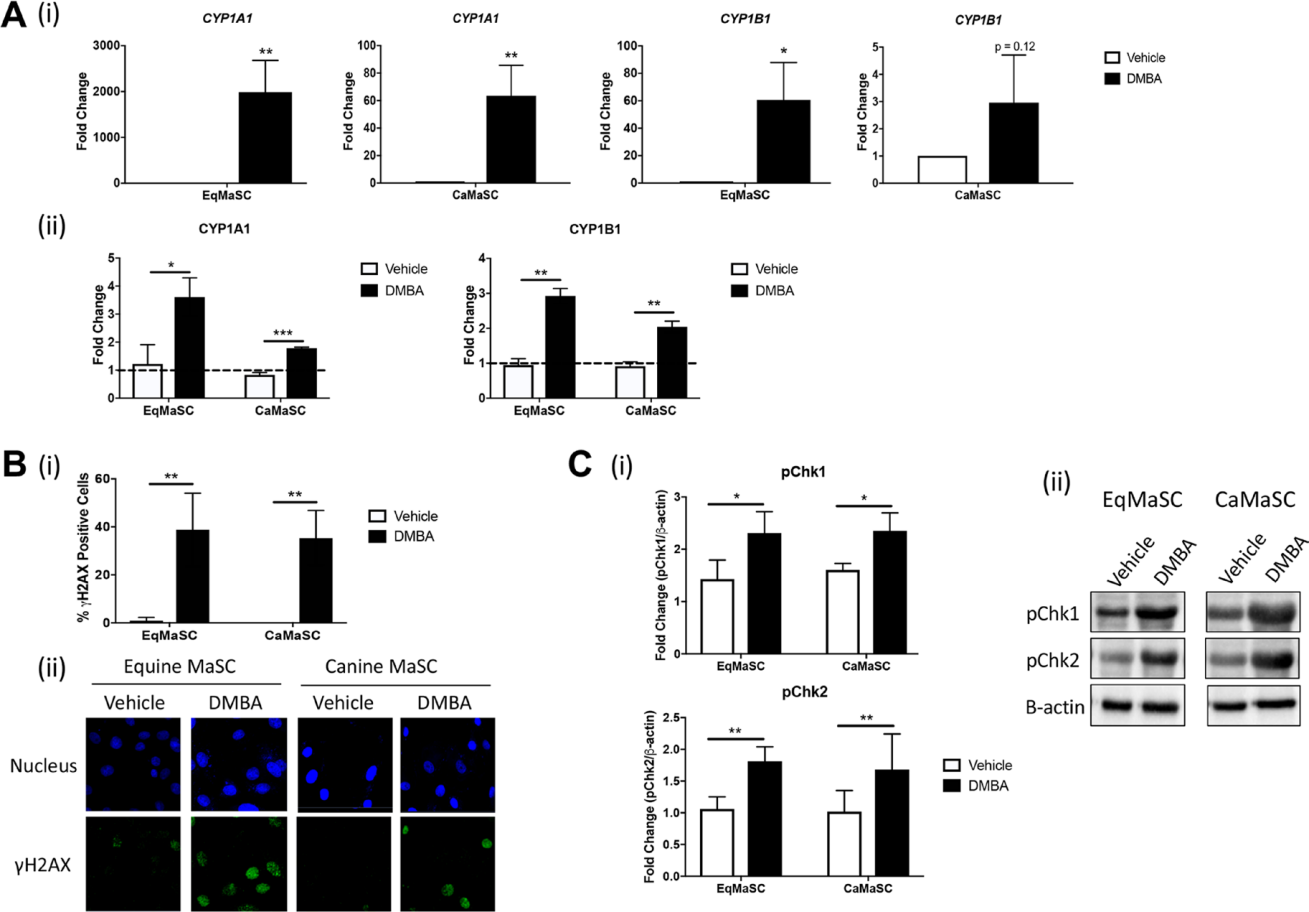
CYP enzymes are activated and DNA damage occurs in response to DMBA treatment in canine and equine MaSC (**A**) Quantification of mRNA expression levels (i) and activity (ii) of CYP1A1 and CYP1B1 in Ca- and EqMaSC after treatment with 5 µM DMBA for 24 h as determined by qRT-PCR and a Promega CYP450 activity assay, respectively. (**B**) Quantification (i) and representative images (ii) of gH2AX positive Ca- and EqMaSC after treatment with 5 µM DMBA for 24 h. Images were taken at 60× magnification. (**C**) Quantification (i) and representative blots (ii) of phosphorylated Chk1 and Chk2 protein expression in Ca- and EqMaSC after treatment with 5 µM DMBA for 24 h. Quantitative data are expressed relative to expression of the loading control β-actin. ^*^p < 0.05, ^**^p < 0.01, ^***^p < 0.001, *n* = 3. Data are presented as the mean ± standard deviation.

Polycyclic aromatic hydrocarbons (PAH), such as 7,12-dimethylbenz[a]anthracene (DMBA), act as carcinogens by binding to DNA after a multi-step metabolic activation via the cytochrome P450 (CYP450) enzymes CYP1A1 and CYP1B1, which ultimately results in the creation of DNA adducts [20]. This sequence of events has been well documented in the mammary gland of rodents and humans; however, no information is available on the activation of DMBA in other mammalian species, including horses and dogs [29]. Therefore, we first explored the effects of DMBA treatment on the expression and activity of CYP1A1 and CYP1B1 in equine and canine mammary stem/progenitor cells (EqMaSC and CaMaSC, respectively). To this end, cells were treated with 5 µM DMBA, a dose previously reported to induce CYP1B1 expression [30], and analyzed 48 h later. qRT-PCR analyses showed that *CYP1A1* expression was significantly upregulated in both species following DMBA treatment (Figure 1A(i)). Likewise, *CYP1B1* expression was also increased, although this did not reach significance in CaMaSC (Figure 1A(i)). Next, luciferase activity assays were used to measure the activity of CYP1A1 and CYP1B1, and the activity of both enzymes was significantly upregulated following DMBA treatment of EqMaSC and CaMaSC (Figure 1A(ii)).

Besides evaluating the activity of DMBA-induced metabolizing enzymes, we also studied DNA damage in MaSC using γH2AX, a prominent marker of DNA double stranded breaks and which is the type of damage caused by DMBA-adducts [20]. Using immunofluorescence (IF), we found a significant increase in γH2AX-positive MaSC from both species upon DMBA treatment, ranging around 42.3% ± 21.2 of γH2AX-positive EqMaSC and 35.3 ± 11.0 of γH2AX-positive CaMaSC (Figure 1B). In addition, we evaluated two checkpoint kinases (Chk), Chk1 and Chk2, which become activated upon DNA damage [31]. Using antibodies specifically recognizing the phosphorylated, active, form of these enzymes and western blot analysis, we observed a significant increase in phosphorylated Chk1 and Chk2 in MaSC from both species upon DMBA treatment, with fold changes of pChk1 of 2.3 ± 0.41 and 2.4 ± 0.34 in EqMaSC and CaMaSC, respectively; and fold changes of pChk2 of 1.81 ± 0.23 and 1.68 ± 0.56 in EqMaSC and CaMaSC, respectively (Figure 1C).

Collectively, these results indicate that MaSC from equine and canine origin react similarly to DMBA exposure by undergoing DNA damage, which is in line with what has previously been reported for human and rodent species.

### DMBA significantly decreases viability in equine, but not canine, MaSC

**Figure 2:**
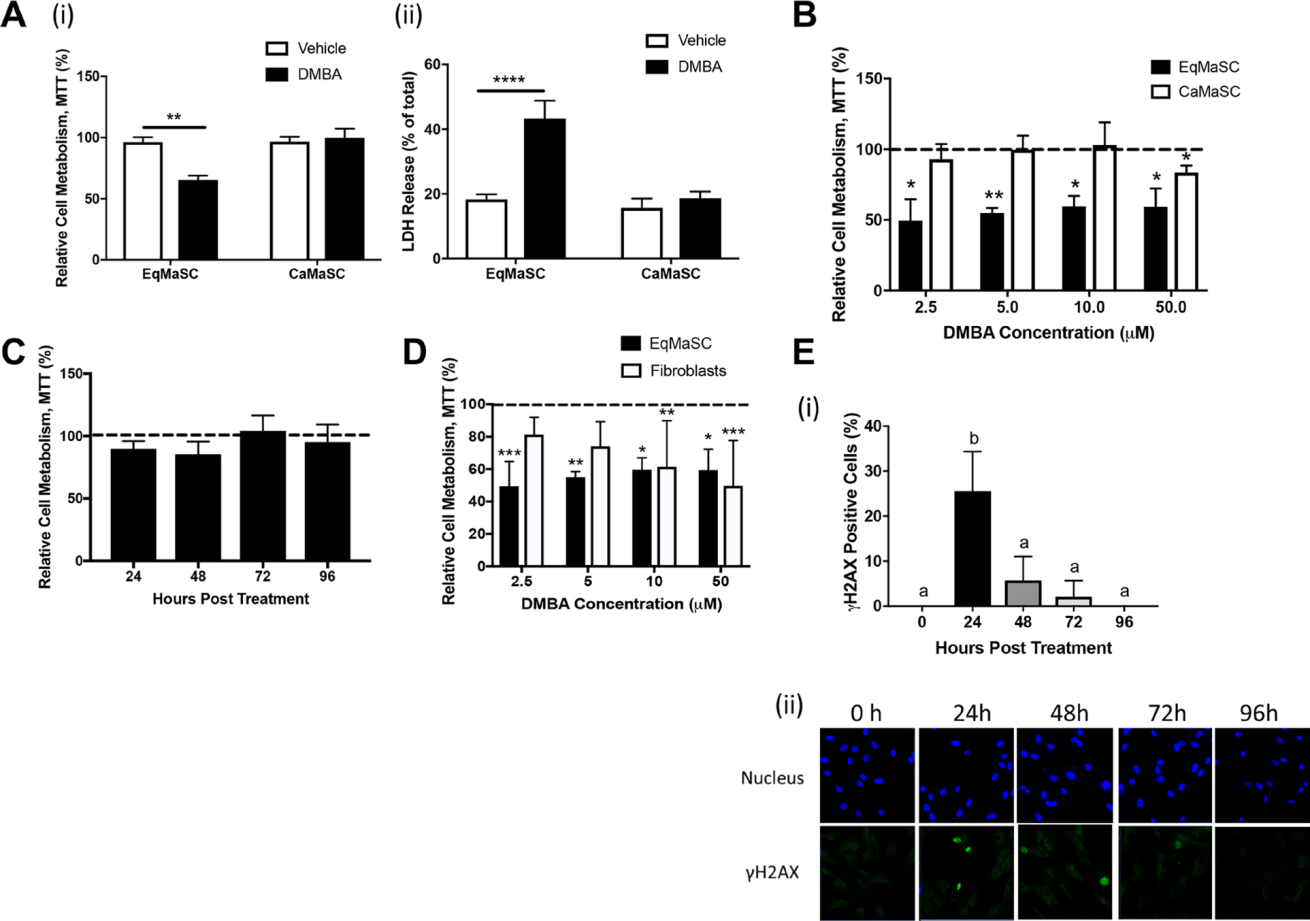
Equine MaSC are more susceptible to DMBA treatment compared to canine MaSC (**A**) Ca- and EqMaSC were treated with 5 µM DMBA for 48 h and viability was analyzed by MTT (i) and LDH (ii) assays. (**B**) Ca- and EqMaSC were treated with increasing concentrations of DMBA for 48 h and analyzed by MTT assay. (**C**) CaMaSC were treated with 5 µM DMBA for different time periods (24 up to 96 h) and analyzed by MTT assay. (**D**) Primary equine mammary fibroblasts and EqMaSC were treated with increasing concentrations of DMBA for 48 h and analyzed by MTT assay. (**E**) Quantification (i) and representative images (ii) of gH2AX-positive CaMaSC after treatment with 5 µM DMBA for increasing time periods. Images were taken at 60x magnification. ^*^p < 0.05, ^**^p < 0.01, ^****^p < 0.0001, *n* = 3. Data are presented as the mean ± standard deviation.

Despite the similarities in DMBA metabolism and DMBA-induced DNA damage observed in equine and canine MaSC, a striking difference was noted in the phenotype of these cells in relation to their ability to remain viable after DMBA treatment. In essence, we found that treatment with 5 µM DMBA significantly reduced the viability of EqMaSC, but not CaMaSC (Figure 2A). Specifically, a significant reduction in cell metabolism was observed, using a 3-(4,5-dimethylthiazol-2-yl)-2,5-diphenyltetrazolium bromide (MTT) assay (Figure 2A(i)), and a significant increase in lactate dehydrogenase (LDH), a cytosolic enzyme that is an indicator of cellular toxicity, was observed, using an LDH release assay (Figure 2A(ii)), in DMBA-treated EqMaSC. In contrast, no effect on cell metabolism or LDH release was observed in DMBA-treated CaMaSC (Figure 2A).

To determine whether this result was dose-dependent, Eq- and CaMaSC were treated with different concentrations of DMBA, varying between 2.5 and 50 μM, for 48 h. A significant reduction in cell viability was observed in EqMaSC treated with every dose of DMBA, whereas CaMaSC started showing some reduced viability at a DMBA concentration of 50 μM, which is 10× higher than the 5 μM concentration used in our experiments (Figure 2B). Moreover, when keeping the concentration of DMBA constant at 5 μM but extending the treatment time, we found that DMBA-treated CaMaSC remained viable over time, even up to 96 h post DMBA treatment (end of experiment) (Figure 2C). Interestingly, when primary equine mammary fibroblasts were treated with these same concentrations, cells were not affected by 2.5 or 5 μM of DMBA, indicating that the observed DMBA effects are specific towards the MaSC population and not just merely reflecting an inherent difference in apoptosis susceptibility between cells from the mammary gland (Figure 2D). Collectively, these results prompted us to hypothesize that CaMaSC, but not EqMaSC, were undergoing DNA damage repair in response to DMBA-induced DNA damage. To evaluate this, we repeated the time course experiment with CaMaSC and performed γH2AX staining to evaluate DNA damage over time. After the initial increase in γH2AX-positive CaMaSC at 24 h after DMBA treatment to 25.5% ± 8.8, which is in line with what we previously observed (Figure 1B), the number of γH2AX-positive cells was significantly less by 48 h and steadily decreased further over time (Figure 2E). These results indicate that CaMaSC indeed repair their DMBA-induced DNA damage, or alternatively, that there are overall less, but viable, cells in the population over time.

### DMBA treatment induces transcriptional changes in equine, but not canine, MaSC

**Figure 3:**
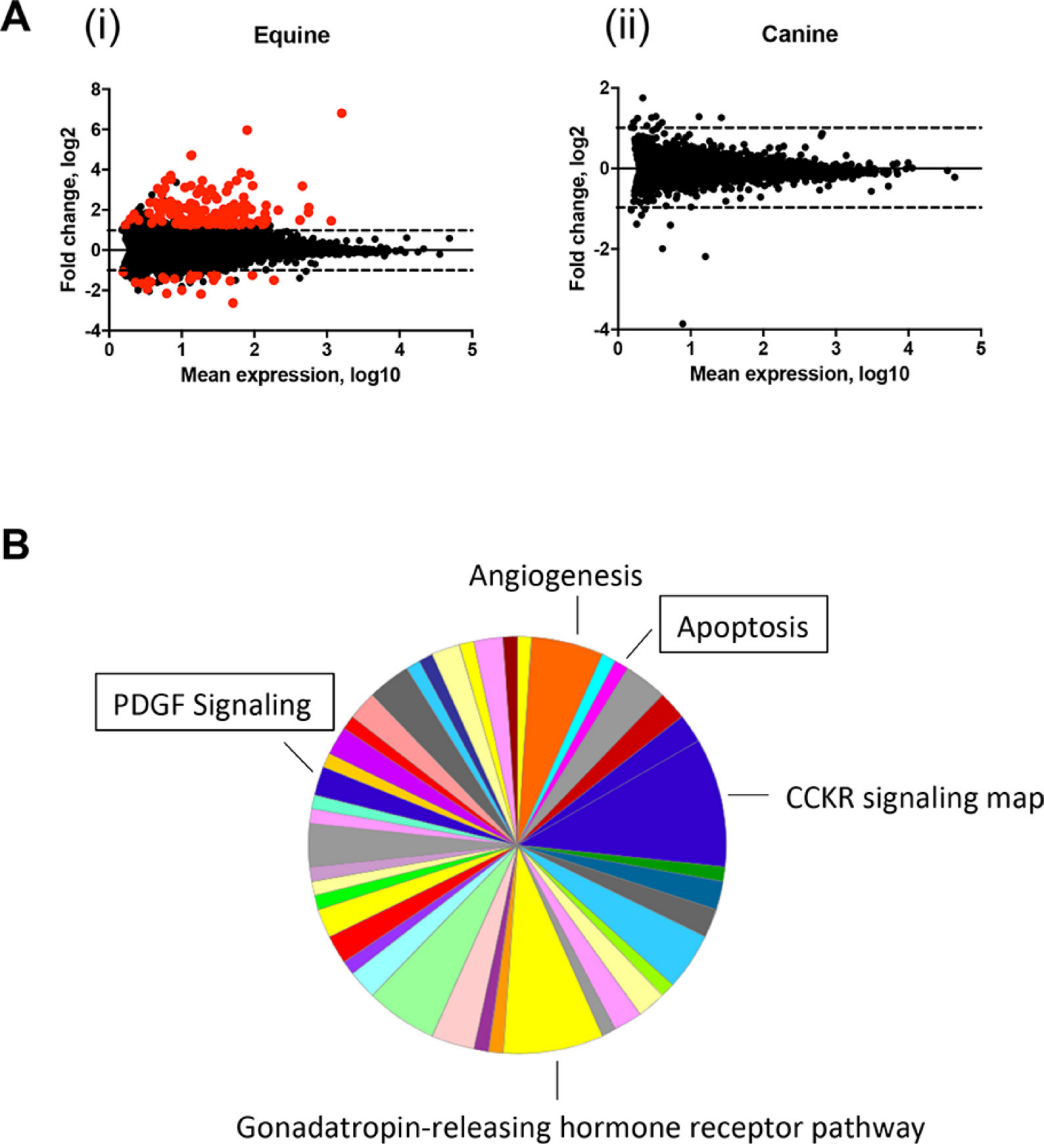
Apoptosis and PDGF signaling pathways are upregulated in equine MaSC following DMBA treatment (**A**) MA analysis showing differentially expressed genes (DEGs) in EqMaSC (i) and CaMaSC (ii) that are either vehicle treated (DMSO) or treated with 5 µM DMBA (*p* < 10^−6^). DEGs are highlighted in red. (**B**) Panther pie chart of the biological pathways represented by upregulated DEGs in EqMaSC in response to DMBA treatment. Boxed pathways were selected for further follow-up studies.

To better characterize the effects of DMBA on MaSC from equine and canine origin on a molecular level, we decided to perform genome-wide transcriptome profiling. To this end, MaSC samples from three individual animals per species were treated with either vehicle (DMSO) or DMBA for 4 h and submitted for RNA deep sequencing. The 4 h treatment was selected to capture a time point before any changes in MaSC viability from either species were observed (data not shown). In DMBA-treated EqMaSC, 172 genes were identified as differentially expressed, with 147 being upregulated and 25 being downregulated, when compared to vehicle-treated cells (Figure 3A(i)). The top 20 up- and downregulated differentially expressed genes (DEGs) are presented in Table 1, and the complete list can be found in Supplementary Table 1. Changes were observed in several genes related to DMBA metabolism, including *ARNTL*, *CYP1A1,* and *CYP1B1*, as well as related to the NOTCH signaling pathway, such as *NOTCH1*, *JAG1*, and *HEY2*. Additionally, genes involved in hormone regulation, such as *RERG*, and in cell cycle regulation, such as *CDKN2A*, were also differentially expressed (Table 1 and Supplementary Table 1). In contrast, and to our surprise, no genes were differentially expressed in DMBA- versus vehicle-treated CaMaSC (Figure 3A(ii)). We then started to analyze the list of both up- and down-DEGs in EqMaSC using the PANTHER statistical overrepresentation test for Gene Ontology (GO) terms. This analysis showed (i) that only the list of upregulated genes matched to known pathways and (ii) that several pathways were differentially regulated in DMBA-treated EqMaSC compared to vehicle-treated EqMaSC (Figure 3B). Based on the reduced cell viability observed in EqMaSC upon DMBA treatment (Figure 2A and 2B), we decided to follow up on the pathways labeled “apoptosis” and “PDGF signaling”, as these pathways are known to play a role in the intrinsic and extrinsic apoptotic pathway, respectively [32, 33]. Based on the fact that no DEGs related to these pathways were found for CaMaSC, we included CaMaSC as a control to determine specificity of the activation signals observed in EqMaSC.

**Table 1:** Top twenty up and downregulated genes in equine MaSC treated with DMBA as identified by RNA sequencing

Up-regulated		Down-regulated	
Gene Abbreviation	Fold Change	Gene Abbreviation	Fold Change
C8orf46	Infinity	KCNE4	–2.6
CYP1A1	6.8	LRRN3	–2.2
CA2	6.0	RERG	–2.2
SCUBE3	5.7	PRSS35	–2.0
SLC27A6	4.8	SLC40A1	–1.9
ELMOD1	4.5	TANC2	–1.7
IL16	3.9	MECOM	–1.7
SLC16A6	3.8	GLI1	–1.6
EREG	3.7	JADE1	–1.6
PTGS2	3.5	PLEKHA5	–1.5
PROKR2	3.5	MMD	–1.5
APCDD1	3.5	TRPS1	–1.5
GPRC5B	3.5	AHR	–1.5
ADORA1	3.4	CCL2	–1.5
DMRTA1	3.2	ZNF503	–1.5
TIPARP	3.2	AIM1	–1.4
GPNMB	3.2	ENSECAG00000016873	–1.4
CYP1B1	3.2	IL34	–1.4
ENSECAG00000022525	3.1	ADAMTS5	–1.4
KIAA1199	3.1	SLC20A2	–1.3

### DMBA treatment activates the intrinsic apoptotic pathway in equine MaSC

**Figure 4:**
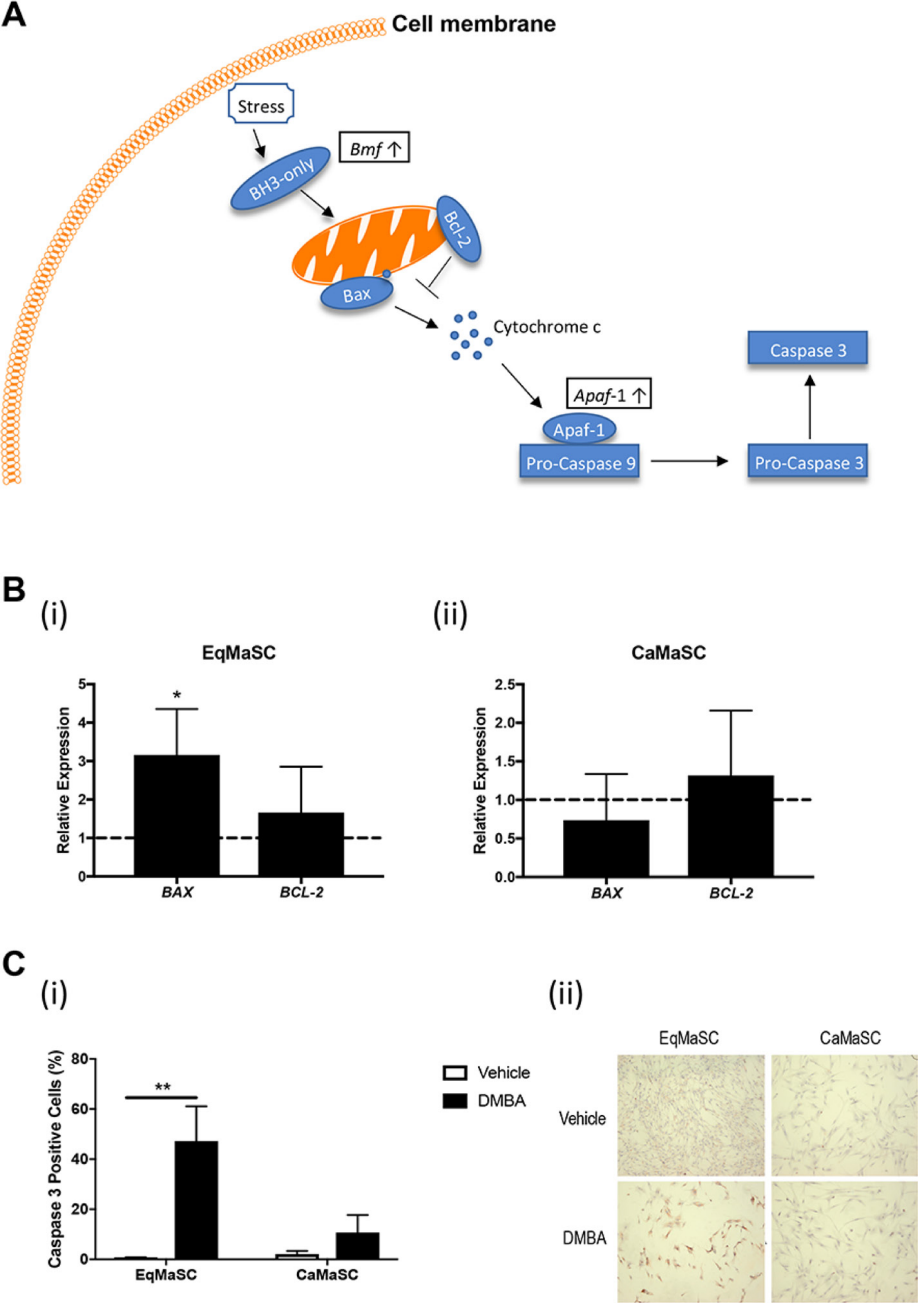
The intrinsic apoptotic pathway is activated in equine MaSC following DMBA treatment (**A**) Schematic representation of important steps during the activation of the intrinsic apoptotic pathway. Boxed genes are significantly upregulated genes as identified by RNAseq. Bcl-2: apoptosis regulator Bcl-2; Bax: apoptosis regulator BAX; Apaf-1: apoptotic protease-activating factor 1. (**B**) Expression of the genes *Bax* and *Bcl-2* in EqMaSC (i) and CaMaSC (ii) after treatment with 5 µM DMBA for 24 h, as determined by qRT-PCR. (**C**) Quantification (i) and representative images (ii) of activated caspase-3-positive Ca- and EqMaSC after treatment with 5 µM DMBA for 24 h. Images were taken at 4x magnification. ^*^p < 0.05, ^**^p < 0.01, *n* = 3. Data are presented as the mean ± standard deviation.

Figure 4A provides a schematic representation of important steps during the activation of the intrinsic, or mitochondrial, apoptotic pathway. Briefly, this pathway is triggered by diverse cellular stresses, such as for example DNA damage. Upon stress, BH3-only proteins become activated which in turn activate Bax and neutralize Bcl-2, leading to mitochondrial outer membrane permeabilization. In response, Cytochrome c is released and binds Apaf-1. Apaf-1 then recruits and binds pro-caspase-9, in turn activating pro-caspase-3 and subsequently caspase-3, ultimately leading to apoptosis [32, 34–37]. Our RNA deep sequencing revealed that several genes important in this pathway were upregulated in DMBA-treated EqMaSC, such as Bcl-2-modifying factor (*Bmf)* and apoptotic protease activating factor 1 *(Apaf-1)* (Figure 4A). To further explore potential changes in this pathway in more detail, we first looked at the expression of two genes downstream of *Bmf*, namely *Bax* and *Bcl-2*, in DMBA-treated EqMaSC [35]. Under normal circumstances, Bcl-2 prevents apoptosis by preventing the activation of Bax or binding the activated Bax; however, when their competitive balance is disrupted, apoptosis is initiated [36]. DMBA treatment of EqMaSC resulted in a significant upregulation of *Bax* and no significant changes in *Bcl-2* expression (Figure 4B(i)). As expected, no change in expression of these two genes was observed in DMBA-treated CaMaSC (Figure 4B(ii)). Combining the observed dysregulation of the *Bax*/*Bcl-2* balance with the upregulation of *Apaf1*, we next evaluated the level of apoptosis in DMBA-treated EqMaSC. Performing immunocytochemistry with a cross-reacting antibody against activated caspase-3 showed a significant increase in the number of activated caspase-3-positive EqMaSC, but not CaMaSC, after DMBA treatment (Figure 4C). Collectively, these results indicate that DMBA treatment of EqMaSC activates the intrinsic apoptotic pathway, ultimately leading to apoptotic cells.

### DMBA treatment activates the extrinsic apoptotic pathway in equine MaSC

**Figure 5:**
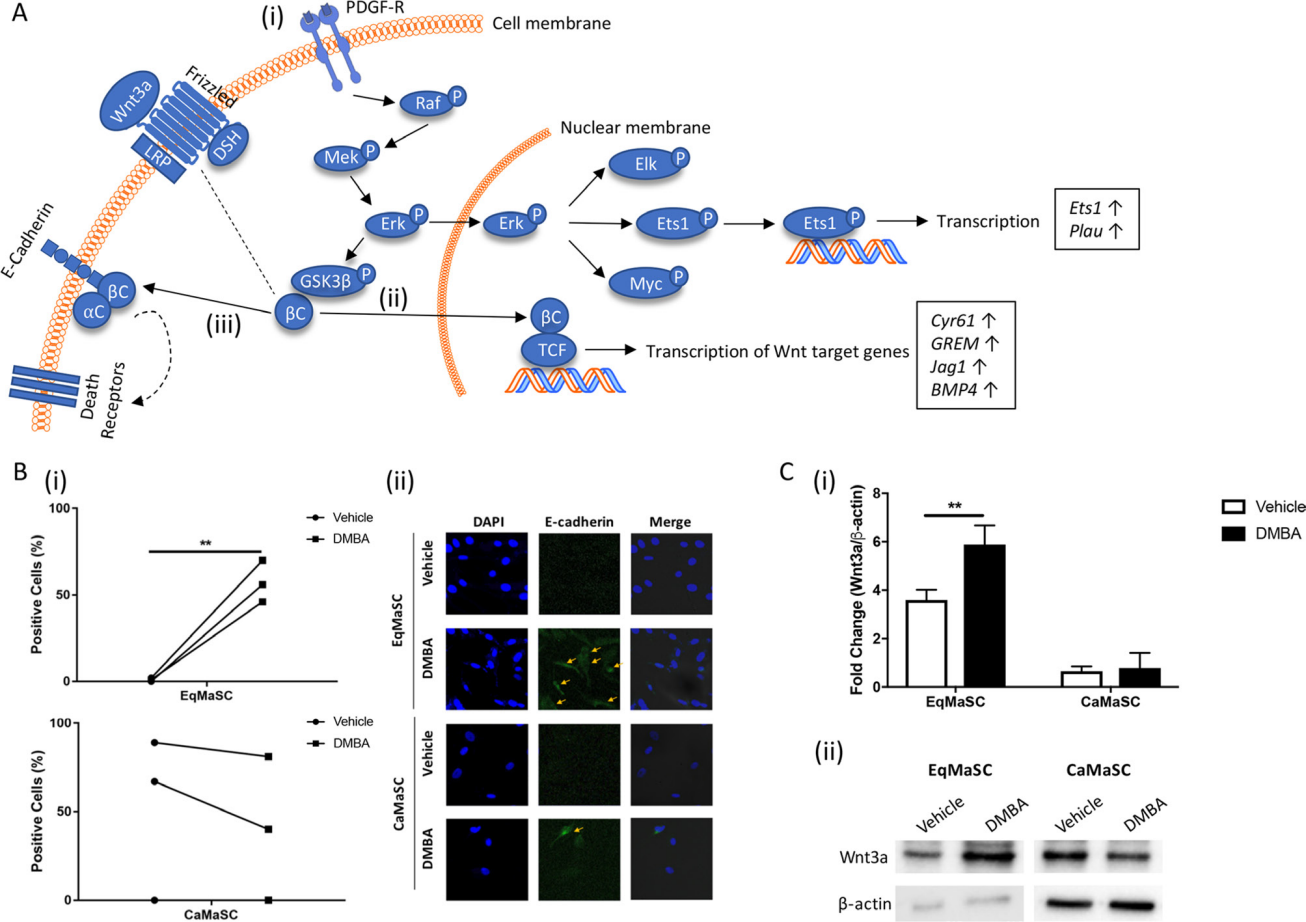
The PDGF signaling pathway is activated in equine MaSC following DMBA treatment (**A**) Schematic representation of important steps during the activation of the extrinsic apoptotic pathway Boxed genes are significantly upregulated genes as identified by RNAseq. aC: α-catenin; bC: β-catenin; LRP: lipoprotein receptor-related protein 5/6; DSH: disheveled; PDGF-R: platelet derived growth factor receptor; Erk: extracellular signal-regulated kinase; GSK3b: glycogen synthase kinase 3 beta; TCF: T-cell factor/lymphoid enhancer factor; Elk: ETS domain-containing protein Elk-1; Ets1: protein C-ets-1; Myc: c-myc; Plau: plasminogen activator urokinase; Cyr61: cysteine-rich angiogenic inducer 61; GREM: glutamate metabotropic receptor 1; Jag 1: jagged 1; BMP4: bone morphogenetic protein 4. (**B**) Quantification (i) and representative images (ii) of E-cadherin-positive Ca- and EqMaSC after treatment with 5 µM DMBA for 24 h. Images were taken at 60× magnification. (**C**) Quantification (i) and representative blots (ii) of Wnt3a protein expression in Ca- and EqMaSC after treatment with 5 µM DMBA for 24 h. Quantitative data are expressed relative to expression of the loading control β-actin. ^**^p < 0.01, *n* = 3. Data are presented as mean ± standard deviation.

Figure 5A provides a schematic, simplified, representation of important steps during the activation of the extrinsic, or death receptor, apoptotic pathway [33, 38]. This complex pathway is triggered by engagement of cell surface ‘death receptors’ with their ligands, and although largely independent from the intrinsic apoptotic pathway, these two pathways do converge at the level of effector caspases [37]. Briefly, dimerization of the Platelet Derived Growth Factor subunit A receptor (PDGF-R) activates the Raf/Mek/Erk signaling cascade, resulting in ERK translocating into the nucleus. This results in the activation of several downstream transcription factors, including ETS domain-containing protein Elk-1 (Elk), protein C-ets-1 (Ets1), and c-Myc (Myc) (Figure 5A(i)). ERK activation also leads to an upregulation of beta catenin (βC) by priming glycogen synthase kinase 3 beta (GSK3b) for inactivation [39]. Upon Wnt activation, βC is translocated into the nucleus where it promotes the transcription of Wnt target genes (Figure 5A(ii)). In addition to its role in the Wnt pathway, βC also binds to and stabilizes E-cadherin [40] (Figure 5A(iii)). This E-cadherin/β-catenin complex can associate with a third factor, α-catenin (αC), to create α catenin-cadherin complex which promotes aggregation of death receptors in the plasma membrane [41]. The clustering of the death receptors increases the sensitivity of the cells to stimuli that promote the extrinsic apoptotic pathway, such as changes in cellular adhesion and morphology [42, 43]. When comparing the upregulated genes in DMBA-treated MaSC, obtained through our RNA deep sequencing analyses, to the intracellular networks activated by PDGF binding, we found that the majority of overlapping genes were involved downstream of Erk activation (Figure 5A). For example, expression of the gene *Ets1*, also known as *p54*, was upregulated (Figure 5A). This transcription factor is activated upon Erk activation and promotes the expression of extracellular matrix proteins, including serine proteases, integrins and E-cadherin [44, 45]. Indeed, our RNA sequencing results also showed an upregulation of plasminogen activator urokinase (*Plau)*, which encodes the serine protease uPA (Figure 5A). To evaluate any changes in E-cadherin protein expression in DMBA-treated EqMaSC, we performed IF using a cross-reacting anti-E-cadherin antibody. E-cadherin expression was significantly increased after DMBA treatment of EqMaSC (Figure 5B(i)). In contrast, and as expected, no significant changes in E-cadherin-positive CaMaSC were found before and after DMBA treatment (Figure 5B(ii)). Besides its role in the extrinsic apoptotic pathway, E-cadherin is also involved in regulating Wnt signaling by promoting the translocation of βC from the cytoplasm to the nucleus, where it activates specific target genes [46]. We decided to follow-up on this pathway also since (i) RNA sequencing found an upregulation of several Wnt target genes, such as cysteine-rich angiogenic inducer 61 (*Cyr61*), glutamate metabotropic receptor 1 (*GREM*), jagged-1 (*Jag1*), and bone morphogenetic protein 4 (*BMP4*), in DMBA-treated EqMaSC (Figure 5A); (ii) we previously demonstrated that EqMaSC have more canonical Wnt signaling than CaMaSC [14]; and (iii) Wnt signaling has many effects on the cell, including a pro-apoptotic effect [47]. Similar to what we reported previously [14], EqMaSC had higher protein levels of Wnt3a, as determined by Western blot analyses, when compared to CaMaSC (Figure 5C). Interestingly, this baseline expression level was significantly increased in EqMaSC, but not CaMaSC, upon DMBA treatment (Figure 5C). Collectively, these results indicate that DMBA treatment of EqMaSC activates the extrinsic apoptotic pathway, via upregulation of several genes that are all involved in stimulating a pro-apoptotic phenotype.

## DISCUSSION

This study was initiated to start exploring potential mechanisms of mammary cancer resistance in the horse. Specifically, we evaluated the intrinsic behavior of MaSC from this mammary cancer-resistant species in response to the chemical 7,12-dimethylbenz[a]anthracene (DMBA) *in vitro* and compared this to the response of MaSC from dogs, a mammary cancer-susceptible species. We found that equine MaSC (EqMaSC), in contrast to canine MaSC (CaMaSC), responded to DMBA-induced DNA damage by inducing apoptosis, a mechanism that allows for the elimination of DNA-damaged MaSC, and hence, would reduce the risk of potential tumor-initiating mutations in this important cell population.

Although polycyclic aromatic hydrocarbons (PAH), such as DMBA, are known to be involved in increased breast cancer incidence [21, 24], the exact underlying mechanisms of how these carcinogens affect the breast, and vice versa, how cells present in the breast respond to PAH, is not fully understood. We chose to study MaSC from canine and equine origin, as these two domestic species share comparable habitats with humans and, consequently, are exposed to many of the same environmental factors as their human companions [48]. The concept of using animals as sentinels for environmental hazards is not new, with the use of canaries to detect carbon monoxide in coal mines and the use of whales to detect environmental contaminants, as well-known historical examples [49, 50].

Interestingly, the apoptotic response of EqMaSC to DMBA-induced DNA damage is similar to what has been described previously for how embryonic and germ line cells respond to irradiation- or chemotherapy-induced DNA damage [51, 52]. Heyer *et al.* demonstrated that irradiation of mouse embryos leads to an elevation in apoptosis in embryonic cells between E6.5 and E7.5, with little effect on somatic cells or later stage embryonic cells [51]. Similarly, Pierpont *et al.* found that after cisplatin-induced DNA damage, testicular germ line cells respond via apoptosis unlike somatic cells [52]. These studies are in line with our result that equine mammary fibroblasts were less affected by low doses of DMBA compared to equine MaSC. Furthermore, similar percentages (between 40–60%) were observed upon DMBA treatment of equine MaSC that were 1) Caspase-3-positive, 2) γH2AX-positive, and 3) had lost their viability. This could suggest that within the MaSC population, only cells that are positive for γH2AX will also be positive for Caspase-3 and, as a result, will lose their viability and die. Lineage tracing experiments coupled with specific MaSC makers for stem and progenitor cells could shed light on which cells within the MaSC population are specifically targeted by DMBA and/or other carcinogen treatments.

In addition, the elephant, a species generally resistant to cancer, was recently reported to initiate apoptosis in cells with DNA damage [2]. Here, peripheral blood lymphocytes were exposed to ionizing radiation to induce DNA damage, and despite having similar levels of γH2AX expression, increased apoptosis was observed in elephant lymphocytes compared to human lymphocytes [2]. Using a candidate-based approach, protein expression of p21, a target of p53, was found to be higher in the elephant compared to human cells. In our study, and based on the RNAseq data, we did not detect differences in expression levels of *p21* or *p53* in either species after DMBA treatment; however, transcriptional changes in genes related to *p21*- and/or *p53*-related signaling pathways may still be occurring. Indeed, we detected changes in gene expression of several genes that are associated with *p53*, including *Ets1*. Previous studies demonstrate that *Ets1* plays a vital role in *p53*-dependent apoptosis [45]. Thus, it is plausible that the genes identified in our present study are also involved in the apoptotic responses observed in cells from cancer-resistant species, such as the elephant, and so it will be interesting to study these molecular mechanisms in more detail across different cancer-resistant species.

Importantly, we wanted to know if the results from our current study were unique to horse and dog or more conserved amongst other mammary cancer-resistant/susceptible species. To preliminary investigate this, we repeated our viability assays with bovine (resistant) and human (susceptible) MaSC after exposure to increasing doses of DMBA. We found that BoMaSC responded similarly to EqMaSC with a significant decrease in viability observed with as little as 2.5 µM DMBA, whereas HuMaSC responded similarly as to what was observed with CaMaSC (Supplementary Figure 1A). These results seem to indicate that the differences in response to DMBA as observed with Eq- and CaMaSC are indeed conserved in other species with varying susceptibility to mammary cancer. Based on these preliminary findings combined with the results we obtained with equine and canine cells, we propose a hypothetical model in which undergoing apoptosis in response to an oncogenic event might contribute to a lower incidence of mammary cancer observed in certain mammalian species. Such a mechanism would allow for the elimination of DNA-damaged MaSC, and hence, reduce the risk of potential tumor-initiating mutations in these cells (Supplementary Figure 1B).

Overall, we strongly believe that using a comparative species approach, such as outlined in our current study, can help to further elucidate mechanisms of cancer resistance, which in the long term could aid in reducing breast cancer risk and/or design of novel approaches for treatment.

## MATERIALS AND METHODS

### Cell culture

Mammary stem/progenitor cells (MaSC) were isolated from equine and canine mammary gland tissues, exactly as previously described [53]. Briefly, equine and canine mammary gland tissues were collected by excising 2 cm^3^ of gland tissue and dissociated mechanically with a sterile scalpel, followed by enzymatic digestion with 0.1% collagenase III (Worthington Biochemical Corporation) at 37° C for 60 min. The resulting cell suspensions were sieved sequentially through sterile 100 µm and 40 µm filters to obtain a single cell suspension. Cells were washed twice in PBS with 1% penicillin/streptomyocin (P/S) by centrifugation at 400 × g and 260 × g for 10 min at RT, respectively. Cells were resuspended in EpSC medium consisting of Dulbecco’s Modified Eagle Medium (DMEM)/F12 (50/50) supplemented with 10% fetal bovine serum (FBS), 2% B27 (all from Invitrogen), 1% P/S (Sigma), 10 ng/mL basic-fibroblast growth factor (BioVision), and 10 ng/mL epidermal growth factor (Sigma). Approximately 5 × 10^5^ cells were seeded on 6-well tissue culture dishes for 1 h, to allow adherence of contaminating fibroblasts, and this was repeated once more. Non-adherent cells were collected and seeded at approximately 20,000 cells/cm^2^ on 6-well ultralow attachment plates (Corning). EpSC medium was refreshed twice a week by means of centrifugation of the cell clusters at 300 × g for 7 min. For further experiments, mammospheres were seeded on adhesive tissue culture flasks in EpSC medium and cultured at 37° C and 5% CO_2_, as previously described [54].

### Luciferase activity assays

Cells were seeded at a density of 5,000 cells/well on a 96 well plate. After 48 h of treatment with either 7,12-Dimethylbenz[a]anthracene (DMBA) or DMSO, a luciferase activity assay for cytochrome p450 (CYP) 1A1 and CYP1B1 was performed according to manufacturer’s instructions (Promega).

### Cell viability assays

Cells were seeded at a density of 5,000 cells/well on a 96 well plate. After 48 h of treatment with either DMBA or DMSO, a 3-(4,5-dimethylthiazol-2-yl)-2,5-diphenyltetrazolium bromide (MTT) *in vitro* toxicology assay (Sigma-Aldrich) and lactate dehydrogenase (LDH) assay (Sigma-Aldrich) were carried out as per manufacturer’s instructions, and absorbance was measured at 570 nm or 490 nm respectively on a Multiskan EX plate reader (Thermo Fisher Scientific). Values were expressed relative to untreated wells.

### RNA sequencing

Cells were cultured for 24 h prior to a 4 h treatment with vehicle (DMSO) or DMBA. Total RNA was extracted with TRIzol reagent following the recommendations of the manufacturer. The quality of total RNA was evaluated using the Agilent 2100 bioanalyzer (Agilent, Palo Alto, CA, USA) with the RNA 6000 Nano LabChip kit. RNA-seq libraries were prepared with the NEBNext Ultra Directional RNA Library Prep Kit (New England Biolabs) using 500 ng total RNA followed by polyA+ enrichment, and were sequenced using Illumina^®^ NextSeq500 (Illumina, San Diego, CA) to obtain 81 nt single-end reads. The reads were trimmed to remove adaptor and low quality bases with cutadapt v1.8.3, aligned with TopHat 2.1.1, and then analyzed for differential gene expression using cuffdiff v2.2.1 2.2.1 using Ensembl annotations (dog: CanFam3; horse: EquCab2). Transcript abundance was measured in fragments per kb of exon per million fragments mapped (FPKM). RNA-seq data have been deposited in the ArrayExpress database at EMBL-EBI (www.ebi.ac.uk/arrayexpress) under accession number E-MTAB-7103.

### Gene expression analyses

Cells were seeded at a density of 2 × 10^5^ in T25 tissue culture flasks. After 24 h, culture medium was removed, cell monolayers were rinsed with phosphate buffered saline (PBS), and cells were incubated in either 5 µM DMBA or DMSO in DMEM for 24 h. Subsequently, mRNA was extracted from the cells using an RNeasy Plus Kit (QIAGEN, Valencia CA) and cDNA was synthesized using M-MLV Reverse Transcriptase (USB, Cleveland, OH), both according to manufacturer’s protocols. SYBER green-based quantitative reverse transcriptase polymerase chain reaction (qRT-PCR) assays were carried out on an Applied Biosystems 7500 Fast Real Time PCR instrument (Applied Biosystems, Carlsbad, CA) to determine fold changes in gene expression. The comparative Ct method was used to quantify gene expression levels where ΔΔCt = ΔCt (sample) – ΔCt (reference). The reference gene Glyceraldehyde 3-phosphate dehydrogenase (GAPDH) was used to normalize canine and feline samples, respectively. Primers to amplify *CYP1A1, CYP1B1, Bax, Bcl-2,* and the housekeeping gene *GAPDH,* were designed using Primer3 software, based on canine and equine sequences found in the National Center of Biotechnology Information (NCBI) GenBank. Primer sequences are listed in Table 2. All samples were run in triplicate.

**Table 2:** Primers used for gene expression analyses

Species	Gene product	Abbreviation	Forward Sequence (5′→3′)	Reverse Sequence (5′→3′)
Canine & Equine	Cytochrome P450, family 1, subfamily A, polypeptide 1	*CYP1A1*	CTTCGTCCCCTTCACCAT	GATCTGCCACTGGTTCAC
Canine & Equine	Cytochrome P450, family 1, subfamily B, polypeptide 1	*CYP1B1*	AGTGGCTGCTCATCCTT	GGCACAAAGCTGGAGAA
Canine	Bcl-2-associated X	*BAX*	GATGAACTGGACAGTAACATGGAG	CAAAGTAGAAGAGGGCAACAACC
Equine	Bcl-2-associated X	*BAX*	CTGAGCAGATCATGAAGACAGG	CTTGAGACACTCGCTCAGCTTC
Canine	B-cell lymphoma 2	*BCL-2*	GTGGATGACTGAGTACCTGAACC	AGACAGCCAGGAGAAGTCAAAC
Equine	B-cell lymphoma 2	*BCL-2*	CCTGTGGATGACTGAATACCTG	GCCAGGAGAAATCAAACAGC
Canine & Equine	Glyceraldehyde 3-phosphate dehydrogenase	*GAPDH*	ACACCCACTCTTCCACCTTC	TACTCCTTGGAGGCCATGTG

### Immunostaining

Cells were treated with 4% formaldehyde, permeabilized with 0.1% Triton X-100, and then blocked with 10% bovine serum albumin (BSA). Cells were incubated with γH2AX (Milipore), Caspase-3 (Abcam), or E-cadherin (Abcam) antibodies, washed with 1% BSA in PBS, and incubated with a 488- or HRP-conjugated secondary antibody (Jackson) and DAPI (Sigma) to label nuclei. Cell images were captured and processed using confocal laser scanning microscope (Zeiss, Oberkochen, Germany).

### Immunoblot analyses

Cells were lysed in RIPA buffer containing 20 mM Tris (pH 8.0), 137 mM NaCl, 10% glycerol, 1% NP-40, 0.1% SDS, 0.5% deoxycholate, and 0.2 mM PMSF and 1X general protease inhibitor. The protein concentrations of whole-cell lysates were determined using the Thermo Fisher BCA assay. 6× sample buffer (300 mM Tris-HCl, pH 6.8, 60% glycerol, 30 mM dithiothreitol, 6% sodium dodecyl sulfate (SDS)) was added to yield a final concentration of 1× and lysates were boiled at 95° C for 10 min. Samples were subjected to SDS/PAGE and transferred to Immobilon PVDF membranes (Millipore) using a transblot turbo system (Biorad). Membranes were blocked in 5% BSA diluted in Tris-buffered saline. The following primary antibodies were incubated overnight at 4° C: anti-Chk1 (R&D), anti-Chk2 (Novus), anti-Wnt3a (R&D), and anti-β-actin (Abcam, loading control). Blots were washed and then incubated with secondary antibodies for 1 h at RT. Blots were washed and visualized by chemiluminescence using Clarity Western ECL (Biorad).

### Statistical analysis

Data were obtained from at least three independent experiments, each using cells from three different individuals, and expressed as the mean ± standard deviation for each group. Statistical analyses, including Student’s *t*-test and one-way analysis of variance, were performed using GraphPad Prism 4.0 software (GraphPad, Inc., La Jolla, CA, USA). *P* < 0.05 was considered to indicate a statistically significant difference.

## SUPPLEMENTARY MATERIALS FIGURE AND TABLE





## Abbreviations

Apaf1: Apoptotic protease-activating factor 1
Bax: Apoptosis regulator BAX
Bcl-2: Apoptosis regulator Bcl-2
BH3: Bcl-2 Homology 3
Bmf: Bcl-2-modifying factor
BMP4: Bone morphogenetic protein 4
BSA: Bovine serum albumin
Chk1/2: Checkpoint kinase 1/2
CYP450: Cytochrome P450
Cyr61: Cysteine-rich angiogenic inducer 61
DEG: Differentially expressed gene
DMBA: 7,12-Dimethylbenz(a)anthracene
Elk1: ETS domain-containing protein Elk-1
Erk: Extracellular signal–regulated kinase
Ets1: Protein C-ets-1
FBS: Fetal bovine serum
GAPDH: Glyceraldehyde 3-phosphate dehydrogenase
GO: Gene Ontology
GREM: Glutamate metabotropic receptor 1
GSK3β: Glycogen synthase kinase 3 Beta
IF: Immunofluorescence
Jag1: Jagged-1
LDH: Lactate Dehydrogenase
MaSC: Mammary Stem/Progenitor Cell
MOMP: Mitochondrial outer membrane potential
MTT: 3-(4,5-dimethylthiazol-2-yl)-2,5-diphenyltetrazolium bromide
Myc: c-Myc
p21: Cyclin-dependent kinase inhibitor 1A
p53: Tumor protein p53
PAH: Polycyclic aromatic hydrocarbon
PBS: Phosphate buffered saline
PDGF: Platelet Derived Growth Factor
subunit A Plau: Plasminogen activator urokinase
P/S: Penicillin/streptomyocin
SDS: Sodium dodecyl sulfate
