# Supplementary material for: Differential signaling pathway activation in 7,12-dimethylbenz[a] anthracene (DMBA)-treated mammary stem/progenitor cells from species with varying mammary cancer incidence
Source: Oncotarget. 2018 Aug 28;9(67):32761–74. doi: 10.18632/oncotarget.25988 (PMC6132353; doi:10.18632/oncotarget.25988)
Supplement: Supplementary file 1 [file oncotarget-09-32761-s001.pdf]

Differential signaling pathway activation in 7,12-dimethylbenz[a] anthracene (DMBA)-treated mammary stem/progenitor cells from species with varying mammary cancer incidence

SUPPLEMENTARY MATERIALS

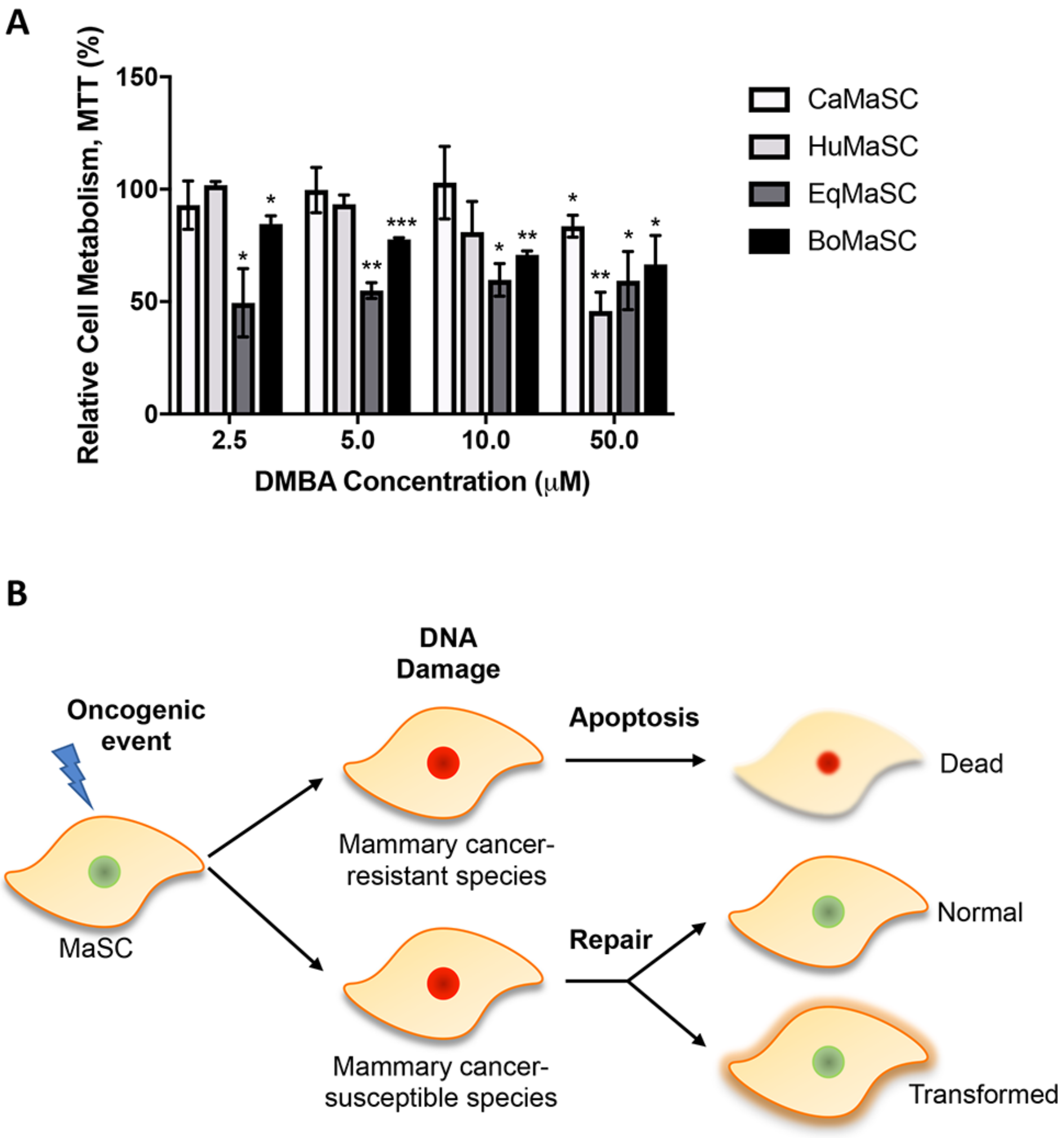

Supplementary Figure 1: Cell death in response to DMBA appears to be conserved across mammary cancer-resistant species. (**A**) Canine, human, equine, and bovine MaSC were treated with increasing concentrations of DMBA for 48 h and analyzed by MTT assay. (**B**) Schematic representation of our working hypothesis describing the differential response of MaSC in response to an oncogenic event, such as DMBA exposure. Following DMBA treatment, MaSC from both mammary cancer-susceptible and -resistant species experience DNA damage. MaSC from mammary cancer-resistant species respond to this damage by initiating an apoptotic response, whereas MaSC from mammary cancer-susceptible species tend to repair the damage. During this repair process, however, mutations may occur that ultimately can lead to mutated stem/progenitor cells and hence, tumorigenesis.

Supplementary Table 1: Complete list of differentially expressed genes (DEGs) following DMBA treatment of EqMaSC.

See Supplementary_Table_1
