# Supplementary material for: Differential signaling pathway activation in 7,12-dimethylbenz[a] anthracene (DMBA)-treated mammary stem/progenitor cells from species with varying mammary cancer incidence
Source: Oncotarget. 2018 Aug 28;9(67):32761–74. doi: 10.18632/oncotarget.25988 (PMC6132353; doi:10.18632/oncotarget.25988)
Supplement: Supplementary file 2 [file oncotarget-09-32761-s002.docx]

**Supplementary Table 1: Complete list of differentially expressed genes (DEGs) following DMBA treatment of EqMaSC**

| **Gene Name** | **p-value** | **Average FPKM DMBA** | **Average FPKM control** | **Fold Change** |
| --- | --- | --- | --- | --- |
| C8orf46 | 0.00005 | 25.5749 | 0.01 | infinity |
| CYP1A1 | 0.00005 | 3173.62 | 28.3878 | 6.8 |
| CA2 | 0.00005 | 157.103 | 2.51803 | 6.0 |
| SCUBE3 | 0.00005 | 13.1015 | 0.257306 | 5.7 |
| SLC27A6 | 0.00005 | 26.4117 | 0.975593 | 4.8 |
| ELMOD1 | 0.00090 | 2.34166 | 0.106134 | 4.5 |
| IL16 | 0.00005 | 3.4472 | 0.235516 | 3.9 |
| SLC16A9 | 0.00005 | 10.9401 | 0.758638 | 3.9 |
| EREG | 0.00005 | 123.711 | 8.63033 | 3.8 |
| PTGS2 | 0.00005 | 159.41 | 11.9747 | 3.7 |
| PROKR2 | 0.00005 | 13.2686 | 1.15935 | 3.5 |
| APCDD1 | 0.00005 | 35.2965 | 3.20845 | 3.5 |
| GPRC5B | 0.00005 | 103.866 | 9.49062 | 3.5 |
| ADORA1 | 0.00045 | 8.32394 | 0.7936 | 3.4 |
| DMRTA1 | 0.00005 | 24.2092 | 2.61812 | 3.2 |
| TIPARP | 0.00005 | 80.1527 | 8.6925 | 3.2 |
| GPNMB | 0.00005 | 170.426 | 18.514 | 3.2 |
| CYP1B1 | 0.00005 | 825.816 | 91.064 | 3.2 |
| ENSECAG00000022525 | 0.00010 | 19.8975 | 2.29181 | 3.1 |
| KIAA1199 | 0.00005 | 10.4969 | 1.24218 | 3.1 |
| KIAA0226L | 0.00005 | 32.0107 | 3.8889 | 3.0 |
| RTN4RL2 | 0.00005 | 45.6883 | 5.55498 | 3.0 |
| AHRR | 0.00005 | 73.4823 | 9.76544 | 2.9 |
| SLC46A2 | 0.00005 | 37.5576 | 5.1836 | 2.9 |
| THEMIS2 | 0.00005 | 28.6411 | 4.06155 | 2.8 |
| SLC10A4 | 0.00005 | 9.3021 | 1.34485 | 2.8 |
| MTSS1 | 0.00005 | 48.9873 | 7.58507 | 2.7 |
| TMEM51 | 0.00005 | 122.967 | 19.8127 | 2.6 |
| KCNE4 | 0.00005 | 14.2561 | 87.9137 | 2.6 |
| ABHD17C | 0.00005 | 52.0232 | 8.91391 | 2.5 |
| BMP4 | 0.00005 | 37.0672 | 6.56791 | 2.5 |
| SHC3 | 0.00005 | 11.7785 | 2.17863 | 2.4 |
| ADAMTS14 | 0.00005 | 2.91493 | 0.546588 | 2.4 |
| ATP2A3 | 0.00005 | 7.30498 | 1.40502 | 2.4 |
| RSAD2 | 0.00105 | 6.13435 | 1.19116 | 2.4 |
| CYP27C1 | 0.00005 | 9.05933 | 1.76644 | 2.4 |
| KLF9 | 0.00005 | 22.1878 | 4.39878 | 2.3 |
| MYH11 | 0.00005 | 7.48855 | 1.49453 | 2.3 |
| SLC1A3 | 0.00005 | 12.7577 | 2.61986 | 2.3 |
| KCNK5 | 0.00005 | 87.6483 | 18.1103 | 2.3 |
| ENSECAG00000024319 | 0.00005 | 118.492 | 24.5183 | 2.3 |
| ABCC5 | 0.00005 | 25.0152 | 5.2338 | 2.3 |
| GCNT1 | 0.00005 | 14.4908 | 3.04991 | 2.2 |
| GREM1 | 0.00005 | 127.795 | 27.0713 | 2.2 |
| ACKR3 | 0.00005 | 239.932 | 51.2391 | 2.2 |
| PAG1 | 0.00005 | 9.26206 | 2.06978 | 2.2 |
| LRRN3 | 0.00005 | 6.6211 | 30.1385 | 2.2 |
| RERG | 0.00115 | 2.2746 | 10.1544 | 2.2 |
| HUNK | 0.00005 | 9.2772 | 2.08546 | 2.2 |
| ETS1 | 0.00005 | 76.1658 | 17.309 | 2.1 |
| IER3 | 0.00005 | 916.992 | 209.783 | 2.1 |
| ABTB2 | 0.00005 | 7.77184 | 1.78844 | 2.1 |
| LPAR1 | 0.00005 | 18.4823 | 4.33315 | 2.1 |
| FAM105A | 0.00020 | 15.5445 | 3.66344 | 2.1 |
| CHST4 | 0.00010 | 13.77 | 3.26945 | 2.1 |
| LYSMD2 | 0.00075 | 25.3527 | 6.07403 | 2.1 |
| KLRG2 | 0.00005 | 86.8171 | 20.8149 | 2.1 |
| ENSECAG00000017406 | 0.00005 | 14.6414 | 3.53012 | 2.1 |
| UGCG | 0.00005 | 111.926 | 27.0705 | 2.0 |
| ANKRD16 | 0.00005 | 60.4412 | 14.7458 | 2.0 |
| CDKN2A | 0.00005 | 144.851 | 35.6967 | 2.0 |
| C19orf38 | 0.00115 | 13.1562 | 3.25879 | 2.0 |
| E2F7 | 0.00005 | 20.8711 | 5.17577 | 2.0 |
| PRSS35 | 0.00005 | 4.0303 | 16.0723 | 2.0 |
| ADRA2A | 0.00005 | 340.358 | 85.6981 | 2.0 |
| NOTCH1 | 0.00005 | 10.5649 | 2.66559 | 2.0 |
| BMF | 0.00005 | 32.9385 | 8.33711 | 2.0 |
| BRI3BP | 0.00005 | 30.9713 | 7.9007 | 2.0 |
| CARD10 | 0.00005 | 88.0499 | 22.5872 | 2.0 |
| SLC40A1 | 0.00005 | 1.3841 | 5.3268 | 1.9 |
| ENSECAG00000017374 | 0.00020 | 26.5507 | 6.92858 | 1.9 |
| LRRC1 | 0.00005 | 11.6925 | 3.11432 | 1.9 |
| LPXN | 0.00005 | 14.2817 | 3.84751 | 1.9 |
| PLAU | 0.00005 | 873.766 | 238.707 | 1.9 |
| TRAM2 | 0.00005 | 63.9942 | 17.6158 | 1.9 |
| BDNF | 0.00005 | 27.5357 | 7.58169 | 1.9 |
| ENSECAG00000008835 | 0.00005 | 5.56737 | 1.5474 | 1.8 |
| EGR1 | 0.00005 | 45.9075 | 12.8716 | 1.8 |
| KLF4 | 0.00005 | 219.079 | 61.9259 | 1.8 |
| ENSECAG00000025166 | 0.00005 | 12.3287 | 3.52781 | 1.8 |
| RHOU | 0.00005 | 83.5631 | 24.0934 | 1.8 |
| CYP2S1 | 0.00005 | 10.9481 | 3.20651 | 1.8 |
| SMAD3 | 0.00005 | 110.655 | 32.5969 | 1.8 |
| NFATC2 | 0.00005 | 7.84037 | 2.31817 | 1.8 |
| TMEM26 | 0.00005 | 53.8227 | 16.1006 | 1.7 |
| TULP4 | 0.00010 | 7.52169 | 2.26732 | 1.7 |
| BHLHE40 | 0.00005 | 72.7867 | 22.7706 | 1.7 |
| RASSF4 | 0.00015 | 21.1752 | 6.62507 | 1.7 |
| TANC2 | 0.00005 | 1.4828 | 4.7757 | 1.7 |
| MECOM | 0.00005 | 1.3682 | 4.3055 | 1.7 |
| MEIS2 | 0.00005 | 26.2024 | 8.42756 | 1.6 |
| ENSECAG00000024818 | 0.00030 | 39.0208 | 12.6167 | 1.6 |
| ENSECAG00000025174 | 0.00005 | 38.065 | 12.3543 | 1.6 |
| BACH2 | 0.00005 | 7.47949 | 2.43797 | 1.6 |
| SYNE3 | 0.00005 | 54.4462 | 17.8679 | 1.6 |
| GLI1 | 0.00055 | 1.1420 | 3.4651 | 1.6 |
| ATF3 | 0.00025 | 14.6657 | 4.83842 | 1.6 |
| CDO1 | 0.00070 | 14.2287 | 4.72035 | 1.6 |
| JADE1 | 0.00030 | 1.8826 | 5.6629 | 1.6 |
| LIPG | 0.00005 | 27.8518 | 9.31414 | 1.6 |
| SMYD2 | 0.00005 | 54.7843 | 18.7321 | 1.5 |
| ENSECAG00000007211 | 0.00010 | 6.33711 | 2.16723 | 1.5 |
| PLEKHA5 | 0.00010 | 1.7856 | 5.1952 | 1.5 |
| MMD | 0.00005 | 23.9240 | 68.9717 | 1.5 |
| RUNX1 | 0.00005 | 29.12 | 10.1163 | 1.5 |
| PRELP | 0.00010 | 39.9272 | 13.9037 | 1.5 |
| ARNTL | 0.00005 | 23.5964 | 8.24641 | 1.5 |
| SLC20A1 | 0.00005 | 220.528 | 77.5522 | 1.5 |
| CHST15 | 0.00050 | 13.8733 | 4.87933 | 1.5 |
| CCDC102A | 0.00005 | 51.3276 | 18.1259 | 1.5 |
| GCLC | 0.00005 | 106.699 | 37.6905 | 1.5 |
| CAMK2G | 0.00005 | 70.9662 | 24.44 | 1.5 |
| HOXC8 | 0.00010 | 54.1515 | 18.659 | 1.5 |
| TRPS1 | 0.00050 | 0.7510 | 2.1223 | 1.5 |
| AHR | 0.00005 | 14.4384 | 40.7502 | 1.5 |
| CCL2 | 0.00005 | 97.7882 | 275.0990 | 1.5 |
| HOXC5 | 0.00005 | 47.5001 | 16.9 | 1.5 |
| GLUL | 0.00005 | 628.082 | 223.475 | 1.5 |
| KCNK2 | 0.00050 | 3.76674 | 1.34045 | 1.5 |
| SMURF1 | 0.00005 | 31.316 | 11.1589 | 1.5 |
| BDKRB2 | 0.00005 | 108.236 | 38.9124 | 1.5 |
| IGFBP-5 | 0.00005 | 74.2494 | 26.7159 | 1.5 |
| CLDND1 | 0.00005 | 188.316 | 67.9926 | 1.5 |
| ENSECAG00000014044 | 0.00005 | 38.6192 | 13.9698 | 1.5 |
| GPRC5A | 0.00065 | 16.625 | 6.02874 | 1.5 |
| ZNF503 | 0.00005 | 6.9047 | 18.9515 | 1.5 |
| ODC1 | 0.00005 | 1676.84 | 613.268 | 1.5 |
| PTHLH | 0.00005 | 21.6898 | 7.93486 | 1.5 |
| GFPT2 | 0.00005 | 166.529 | 60.9946 | 1.4 |
| AIM1 | 0.00010 | 3.1428 | 8.5620 | 1.4 |
| ENSECAG00000016873 | 0.00005 | 4.3252 | 11.5927 | 1.4 |
| IL34 | 0.00115 | 7.4235 | 19.8432 | 1.4 |
| GALNT10 | 0.00005 | 25.8605 | 9.68142 | 1.4 |
| TRAF3IP2 | 0.00005 | 28.5103 | 10.7329 | 1.4 |
| C5orf30 | 0.00015 | 7.79937 | 2.88657 | 1.4 |
| EDG1 | 0.00005 | 67.0102 | 25.778 | 1.4 |
| XYLT1 | 0.00005 | 15.6449 | 6.05162 | 1.4 |
| GSN | 0.00005 | 205.322 | 79.961 | 1.4 |
| ADAMTS5 | 0.00035 | 3.0915 | 7.9259 | 1.4 |
| KCNF1 | 0.00010 | 16.4667 | 6.42718 | 1.4 |
| SRC | 0.00005 | 61.9163 | 24.2674 | 1.4 |
| ARID3B | 0.00080 | 5.54261 | 2.17555 | 1.3 |
| RASL11B | 0.00005 | 126.045 | 49.7266 | 1.3 |
| MICAL2 | 0.00005 | 87.7288 | 34.7491 | 1.3 |
| TIAM2 | 0.00005 | 11.9186 | 4.75143 | 1.3 |
| RIN1 | 0.00005 | 16.741 | 6.69643 | 1.3 |
| DAAM2 | 0.00005 | 96.7106 | 38.7056 | 1.3 |
| SERPINB2 | 0.00005 | 82.5014 | 33.0592 | 1.3 |
| COL5A3 | 0.00005 | 13.4297 | 5.38248 | 1.3 |
| HIP1 | 0.00005 | 41.6702 | 16.7933 | 1.3 |
| SPRY4 | 0.00010 | 35.5304 | 14.3238 | 1.3 |
| FHL1 | 0.00045 | 23.3398 | 9.4164 | 1.3 |
| PLK3 | 0.00010 | 19.5339 | 7.90796 | 1.3 |
| ISOC1 | 0.00005 | 193.754 | 78.5539 | 1.3 |
| KIAA1522 | 0.00005 | 17.4433 | 7.30502 | 1.3 |
| ZHX3 | 0.00005 | 14.9618 | 6.07965 | 1.3 |
| CYR61 | 0.00005 | 126.123 | 51.4311 | 1.3 |
| PHLDA1 | 0.00005 | 184.856 | 75.7533 | 1.3 |
| EDEM3 | 0.00005 | 21.4702 | 8.82802 | 1.3 |
| SLC20A2 | 0.00005 | 16.6090 | 40.2411 | 1.3 |
| ANGPTL4 | 0.00100 | 17.6641 | 42.7911 | 1.3 |
| TUBB3 | 0.00005 | 55.6138 | 132.1430 | 1.2 |
| PLEKHA1 | 0.00005 | 50.5179 | 21.2636 | 1.2 |
| TRAF1 | 0.00005 | 67.0409 | 28.2667 | 1.2 |
| JAG1 | 0.00005 | 32.9025 | 13.8803 | 1.2 |
| HMGCS1 | 0.00005 | 9.9746 | 23.5615 | 1.2 |
| NFATC4 | 0.00005 | 55.3112 | 23.4803 | 1.2 |
| HEY2 | 0.00005 | 90.2194 | 38.5803 | 1.2 |
| GSAP | 0.00005 | 14.6386 | 6.26467 | 1.2 |
| DAPK1 | 0.00005 | 18.021 | 7.71415 | 1.2 |
| ENSECAG00000002353 | 0.00055 | 15.6503 | 36.4393 | 1.2 |
| TBX2 | 0.00025 | 8.26155 | 19.2328 | 1.2 |

Fold changes were calculated by comparison of the mean read per kilobase of transcript per million (FPKM) values of DMSO (control) vs DMBA treated cells at 4 h post treatment. Genes were ranked according to the absolute value of the fold change.
